# Improved sensitivity and precision in multicentre diffusion MRI network analysis using thresholding and harmonization

**DOI:** 10.1016/j.nicl.2022.103217

**Published:** 2022-10-03

**Authors:** Bruno M. de Brito Robalo, Alberto de Luca, Christopher Chen, Anna Dewenter, Marco Duering, Saima Hilal, Huiberdina L. Koek, Anna Kopczak, Bonnie Yin Ka Lam, Alexander Leemans, Vincent Mok, Laurien P. Onkenhout, Hilde van den Brink, Geert Jan Biessels

**Affiliations:** a Department of Neurology and Neurosurgery, UMC Utrecht Brain Center, University Medical Center Utrecht, Utrecht, the Netherlands; b Image Sciences Institute, University Medical Center Utrecht, Utrecht University, Utrecht, the Netherlands; c Memory, Aging and Cognition Center, Department of Pharmacology, Yong Loo Lin School of Medicine, National University of Singapore, Singapore; d Institute for Stroke and Dementia Research (ISD), University Hospital, LMU Munich, Germany; e Medical Image Analysis Center (MIAC AG) and Department of Biomedical Engineering, University of Basel, Basel, Switzerland; f Saw Swee Hock School of Public Health, National University of Singapore and National University Health System, Singapore; g Department of Geriatric Medicine, University Medical Center Utrecht, Utrecht, the Netherlands; h Division of Neurology, Department of Medicine and Therapeutics, Gerald Choa Neuroscience Centre, Faculty of Medicine, Prince of Wales Hospital, The Chinese University of Hong Kong, Shatin, Hong Kong Special Administrative Region

**Keywords:** Connectivity, Diffusion MRI, Thresholding, Harmonization: cerebral small vessel disease, White matter

## Abstract

**Purpose:**

To investigate if network thresholding and raw data harmonization improve consistency of diffusion MRI (dMRI)-based brain networks while also increasing precision and sensitivity to detect disease effects in multicentre datasets.

**Methods:**

Brain networks were reconstructed from dMRI of five samples with cerebral small vessel disease (SVD; 629 patients, 166 controls), as a clinically relevant exemplar condition for studies on network integrity. We evaluated consistency of network architecture in age-matched controls, by calculating cross-site differences in connection probability and fractional anisotropy (FA). Subsequently we evaluated precision and sensitivity to disease effects by identifying connections with low FA in sporadic SVD patients relative to controls, using more severely affected patients with a pure form of genetically defined SVD as reference.

**Results:**

In controls, thresholding and harmonization improved consistency of network architecture, minimizing cross-site differences in connection probability and FA. In patients relative to controls, thresholding improved precision to detect disrupted connections by removing false positive connections (precision, before: 0.09–0.19; after: 0.38–0.70). Before harmonization, sensitivity was low within individual sites, with few connections surviving multiple testing correction (k = 0–25 connections). Harmonization and pooling improved sensitivity (k = 38), while also achieving higher precision when combined with thresholding (0.97).

**Conclusion:**

We demonstrated that network consistency, precision and sensitivity to detect disease effects in SVD are improved by thresholding and harmonization. We recommend introducing these techniques to leverage large existing multicentre datasets to better understand the impact of disease on brain networks.

## Highlights

• Harmonization with rotation invariant spherical harmonic (RISH) features and network thresholding improve cross-site consistency of dMRI-based brain networks.
• RISH harmonization enables multicentre data pooling to increase sample size and infer patterns of network injury with improved sensitivity.
• When network thresholding is applied in combination with harmonization, false positive connections are removed from the network, thus improving the precision of the detected patterns of injury.

## 1 Introduction

Describing the human brain as a network of nodes (gray matter regions) connected by edges (white matter pathways) can provide powerful insights into neurological disorders (Bullmore and Sporns, 2009, van den Heuvel and Sporns, 2013). Changes in local and global properties of brain networks reconstructed from diffusion MRI (dMRI) have been associated with many disease processes (Pievani et al., 2014). An important issue in this rapidly evolving research field is that network analysis is highly dependent on how the network nodes and connections are defined, particularly when trying to identify specific connections or subnetworks that are disrupted by particular disease. To date, network studies have used various definitions for edges and nodes, different tractography algorithms and edge-weights, resulting in inconsistent reported architectures across datasets (for example, Du et al., 2020, Tuladhar et al., 2017).

Without proper processing, brain networks reconstructed from dMRI are known to contain many false-positive connections which contribute for variability in network architecture (Buchanan et al., 2014). This can be alleviated by thresholding the network to a core structure with more reliable connections, over which eventual disease effects can be assessed with higher confidence (McColgan et al., 2018). A second issue that needs to be considered when detecting network injury patterns in relation to disease is statistical power. To identify specific disrupted connections, methods such as network-based statistics (NBS) are applied to fit a univariate model at every connection and test hypotheses of interest (e.g., differences in fractional anisotropy – FA – between patients and controls at connection level) (Zalesky et al., 2010). Since networks typically contain thousands of connections, multiple tests are performed, often in study populations with a modest sample size, resulting in type I and type II errors. In this scenario, filtering for potentially false-positive connections reduces multiple testing. Moreover, an obvious strategy to improve power is to increase the sample size by pooling existing data from different sites. To date, however, dMRI data pooling has been hampered by acquisition-related differences across sites (Bonilha et al., 2015, Vollmar et al., 2010). As a consequence, network studies of network topology in patients have been predominately single centre with inconsistent recognition of network connections affected by disease (Du et al., 2020, Reijmer et al., 2016, Tuladhar et al., 2017).

Here, we address the challenge of using multicentre dMRI data to study abnormalities in network topology, using cerebral small vessel disease (SVD) as an exemplar condition. In this context SVD is of clear interest because it is considered to affect cognition by disrupting brain connectivity as a result of vascular injury (Frey et al., 2021, Lawrence et al., 2018, Reijmer et al., 2015). However, the exact impact of SVD on network topology is still unknown, with variable results between studies (Du et al., 2020, Tuladhar et al., 2017). This likely relates to the issues raised above, in particular possible confounding by false positive connections and insufficient sample sizes. Recently, in a monocentre setting, we demonstrated the benefits of pre-processing steps such as thresholding to reduce the number of false positive connections and generate more consistent network architectures to assess disease effects (de Brito Robalo et al., 2020). We have also shown proof of concept of raw data harmonization to minimize acquisition-related differences in multicentre dMRI, at the level of basic diffusion metrics (e.g., FA from the white matter skeleton; de Brito Robalo et al., 2021). In this study, we apply these techniques to higher order diffusion models, to demonstrate the feasibility of multicentre studies of network topology in disease.

We assess the joint application of network thresholding and harmonization to improve sensitivity and precision to detect connections disrupted in SVD. We expect that applying network thresholding leads to more consistent architectures by reducing false positives and improving precision in network analysis, whereas dMRI harmonization improves the sensitivity, by reducing site variability to leverage large multicentre data to increase sample size. We reconstruct dMRI-based networks from four samples with sporadic SVD and one sample with a monogenic form of SVD (Cerebral autosomal dominant arteriopathy with subcortical infarcts and leukoencephalopathy – CADASIL, Craggs et al., 2014) and controls. We first evaluate cross-site consistency of network architecture before and after thresholding and harmonization, using age-matched controls from each site. Next, we determine within sites which connections are most affected in patients relative to controls, before and after thresholding and harmonization. To date there is no gold standard for which connections are actually affected by SVD. We therefore use the genetically defined SVD (CADASIL), which is characterized by severe and pure vascular injury, as a reference standard to determine precision and sensitivity for detecting affected connections in the sporadic SVD samples. Finally, we assessed how pooling of scans from all sporadic SVD samples, before and after thresholding and harmonization, improved sensitivity and precision to detect affected connections.

## 2 Methods

### 2.1 Datasets

Four samples of patients with sporadic SVD and one sample with CADASIL were included. Inclusion and exclusion criteria of each cohort are reported in the original studies (summarized below). For the current analysis, we used a harmonized definition for selecting patients and controls from the original cohorts, which was based primarily on the degree of white matter injury, since our objective was to assess benefits of thresholding and harmonization on the detection of injured connections but not their functional impact. Patients had symptomatic SVD defined as a) history of stroke, with a corresponding small subcortical infarct visible on MRI or b) cognitive complaints and presence of white matter hyperintensity (WMH) burden on MRI (Fazekas score ≥ 2, Fazekas et al., 1987). Patients were excluded if they had other major neurological or psychiatric conditions (e.g., multiple sclerosis, epilepsy, Parkinson’s disease). Each cohort also included control subjects, with no history of stroke or cognitive complaints and no signs of lacunes on MRI and minimal WMH (Fazekas score 0 or 1). All subjects had T1-weighted and dMRI scans. Characteristics of the study samples (629 patients and 166 controls in total) are provided in supplementary Table 1. All studies included in this analysis were approved by the ethics committees of the respective institutions and all participants provided written informed consent.

**Table 1:** Precision to detect connections affected in sporadic SVD samples and pooled data, relative to the reference CADASIL sample.

		**UT**	**ZO**	**HK**	**SI**	**Pooled data (non-harmonized)**	**Pooled data (harmonized)**
**Connections with large effect sizes (>0.8)**	TP	56	105	30	63	231	67
	FP	576	588	350	270	2040	1065
	**Precision**	**0.09**	**0.15**	**0.08**	**0.19**	**0.10**	**0.06**
**Survive Thresholding**	TP	54	100	23	56	206	62
	FP	30	83	38	38	88	11
	**Precision**	**0.64**	**0.55**	**0.38**	**0.59**	**0.70**	**0.85**
**Survive Thresholding and FDR**	TP	17	0	0	0	140	37
	FP	8	0	0	0	24	1
	**Precision**	**0.68**	N/A	N/A	N/A	**0.85**	**0.97**

Abbreviations: TP - true positives; FP – false positives.

**Utrecht:** Patients (n = 170) were selected from a memory clinic cohort (Aalten et al., 2014). Controls (n = 46) were recruited from a community-based cohort (Reijmer et al., 2013). MRI scans were acquired on a 3 Tesla Philips scanner (Achieva, Philips, Best, the Netherlands). T1-weighted images had a voxel size of: 1 × 1 × 1 mm3, echo time (TE): 4.5 ms and repetition time (TR): 7.9 ms. dMRI data were obtained with a voxel size: 2.5 × 2.5 × 2.5 mm3, TR/TE: 6638/73 ms, 45 diffusion gradients directions with a b-value of 1200 s/mm2, and 1b = 0 s/mm2 averaged 3 times.

**Utrecht 2 (ZOOM):** A second dataset from the University Medical Center Utrecht consisted of patients (n = 26) and controls (n = 18) from an ongoing prospective observational cohort study (ZOOM@SVDs, van den Brink et al., 2021). MRI scans were acquired using the same scanner system and acquisition parameters as “Utrecht”, albeit with different scanner software versions. Utrecht2 is referred to as “ZOOM” for the remainder of this manuscript.

**Hong Kong:** Subjects from a community-based cohort that fitted our definition of patients (n = 20) and controls (n = 20) were included (Lam et al., 2019). MRI scans were acquired on a 3 Tesla Philips scanner (Achieva, Philips, Best, the Netherlands). T1-weighted images were obtained with a voxel size: 0.60 × 1.04 × 1.04 mm^3^, TR/TE: 7.49/3.46 ms and dMRI had a TR/TE: 8944/60 ms, voxel size: 1 × 1 × 2 mm3; 32 diffusion gradient directions with b-value 1000 s/mm2 and 1b = 0 s/mm2.

**Singapore:** A second community-based cohort was included, with cases (n = 359) and controls (n = 54) fitting our selection criteria (Hilal et al., 2013). MRI were performed on a 3 Tesla Siemens Magnetom Trio Tim scanner (Siemens Healthineers, Erlangen, Germany). T1-weighted images had TR/TE: 2300/1.9 ms, voxel size: 1 × 1 × 1 mm^3^ and dMRI was acquired with a TR/TE: 6800/85 ms, voxel size: 3.1 × 3.1 × 3 mm^3^; 61 diffusion gradient directions with b-value 1150 s/mm^2^ and 7b = 0 s/mm^2^.

**Munich**: Patients (n = 54) with CADASIL and controls (n = 28) were selected from a prospective study (Baykara et al., 2016). All MRI scans were acquired on a 3 Tesla Magnetom Verio scanner (Siemens Healthineers, Erlangen, Germany). T1-weighted images were obtained using TR/TE: 2500/4.73 ms, voxel size: 1 × 1 × 1 mm^3^ and dMRI were acquired with a voxel size: 2 × 2 × 2 mm^3^, TR/TE: 12,700/81 ms, 30 diffusion gradient directions with a b-value of 1000 s/mm^2^, and 1b = 0 s/mm^2^.

### 2.2 Network reconstruction

Diffusion scans were pre-processed using ExploreDTI version 4.8.6 (Leemans et al., 2009). Images were corrected for signal drift (Vos et al., 2017), eddy currents, subject motion with rotation of the B-matrix (Leemans and Jones, 2009), and susceptibility induced distortions (Veraart et al., 2013). Individual T1-weighted images were resampled to an isotropic resolution of 2 mm^3^, then dMRI scans were nonlinearly registered to the resampled T1 images to correct for susceptibility artefacts. Subsequently, the diffusion tensor was estimated with a robust approach (Tax et al., 2015). Deterministic fiber tractography was performed with seed points distributed uniformly throughout the brain, using ExploreDTI. Streamlines were propagated using integration over fiber orientation distributions (FOD), with a step size of 1 mm. FODs were inferred using constrained spherical deconvolution (CSD) with a maximum harmonic order (l-max) of 6 (Jeurissen et al., 2011). Fiber tracking was terminated when streamlines entered a voxel with FOD amplitude < 0.1, or when the deflection was >45°. Streamlines with a length outside of the bounds 10–500 mm were deemed implausible and excluded. To reconstruct whole brain networks, the Automated Anatomical Labeling (AAL) atlas was used to define 90 cortical and subcortical brain regions that represent network nodes (Tzourio-Mazoyer et al., 2002). Networks were reconstructed by combining the segmented AAL regions with the tractography data. Two nodes were considered connected if they contained the end-points of at least one streamline, resulting in 90 × 90 binary connectivity matrices. The connectivity matrices were also weighted by fractional anisotropy (FA) to obtain 90 × 90 FA-weighted connectivity matrices.

### 2.3 Analysis

#### 2.3.1 Cross-site consistency of network architecture

We assess whether harmonization and thresholding improve the consistency of network architecture across sites. For this objective we focused on networks of controls, which were age-matched across sites (n = 15 for each site). The most straightforward definitions of network architecture are the presence or absence of connections (i.e., the binary structure) and their corresponding weights (e.g., FA). These characteristics constitute the basis of all network properties from which all network metrics are derived (Messaritaki et al., 2019). To evaluate consistency of the binary structure, we first constructed a group-level probability matrix for each site by averaging the binary connectivity matrices of all subjects. In this probability matrix, each entry represents the probability a connection being reconstructed in that group. For example, a connection with a probability of 0.5 is detected in 50 % of subjects. Second, we defined consistency between two sites as the average relative difference (in %) between the group-level probability matrices.

(1) $$\mathrm{difference}\;=100\%\;\times \frac{\;|Site1-Site2|}{\frac{Site1+\;Site2}{2}}\;\;\;\;$$

Note that the lower the difference between sites the higher the consistency in connection probability. To evaluate consistency of FA-weights, we also constructed a group group-level matrix containing the average FA of connections reconstructed within each group. We computed the consistency in FA as the difference between the group-level FA-matrices of different sites as by the formula above. We determined the consistency before and after thresholding and harmonization, with each method being applied separately and in combination.

Thresholding was performed on the group-level probability matrix by removing connections with low probability until a fixed network density of 15 % was achieved (de Reus and van den Heuvel, 2013a, de Reus and van den Heuvel, 2013b, Roberts et al., 2017). This thresholding approach removes the most improbable connections within each site, while also ensuring that networks with equal densities are achieved across all sites.

Harmonization was performed by scaling the dMRI signal off 15 age and sex-matched controls of all sites to an arbitrary reference (Utrecht) using rotation invariant spherical harmonic (RISH) features (Cetin Karayumak et al., 2019). Both our previous work (de Brito Robalo et al., 2021) and other method papers (Mirzaalian et al., 2015, Cetin Karayumak et al., 2019) have shown that 15 to 20 controls is sufficient to calculate average RISH features that capture group properties of each site, rather than individual characteristics, to perform effective harmonization. This harmonization method is applied to the raw dMRI signal to remove acquisition-related differences while preserving biological effects and between-subject variation.

#### 2.3.2 Sensitivity and precision to disease effects in SVD

Here we assess if thresholding and harmonization improve precision and sensitivity to detect connections affected in patients with SVD. Clearly there is no established ground-truth, apart from histology, on which tracts are preferentially affected by SVD and which are relatively spared (Craggs et al., 2014). Moreover, substantial inter-individual variation is likely to occur, according to lesion burden and location. Yet, we required a reference standard to provide a basis to test the validity of our approach. For this we used a sample of patients with a monogenic form of SVD (CADASIL) with a high burden of disease, as a pure form of SVD to which we could compare the findings in the other samples with sporadic SVD (Di Donato et al., 2017). To identify connections most affected in SVD, network-based statistics adjusted for age and sex was performed to compare the FA of each connection between patients and controls. Specifically, two-sample t-tests were performed for each connection and False Discorvery Rate (FDR) correction with 50 thousand permutations was used to account for multiple comparisons. Connections with large effect sizes (Cohen’s d > 0.8) relative to controls that survive network thresholding and FDR in the CADASIL cohort were used as reference standard of SVD-related patterns of network injury.

To estimate precision and sensitivity within each sporadic SVD cohort, NBS with the same parameters as described above was performed to detect connections strongly affected in patients relative to controls. These affected connections were compared to those found in the CADASIL reference. Precision was defined as the ratio between true positives (TP; i.e., connections also affected in the reference standard) and the total number of connections detected:

(2) $$\mathrm{precision}\;=\frac{\mathrm{TP}}{\mathrm{TP}+\;FP\;}\;\;\;\;$$

here, false positives (FP) are connections detected in sporadic SVD but not in the reference. Precision was estimated for the pooled dataset and all sporadic SVD cohort, before and after harmonization and thresholding. The number of connections with large effect size that survive thresholding and FDR-correction was used as an indicator of sensitivity.

## 3 Results

### 3.1 Cross-site consistency of network architecture

**Fig. 1:**
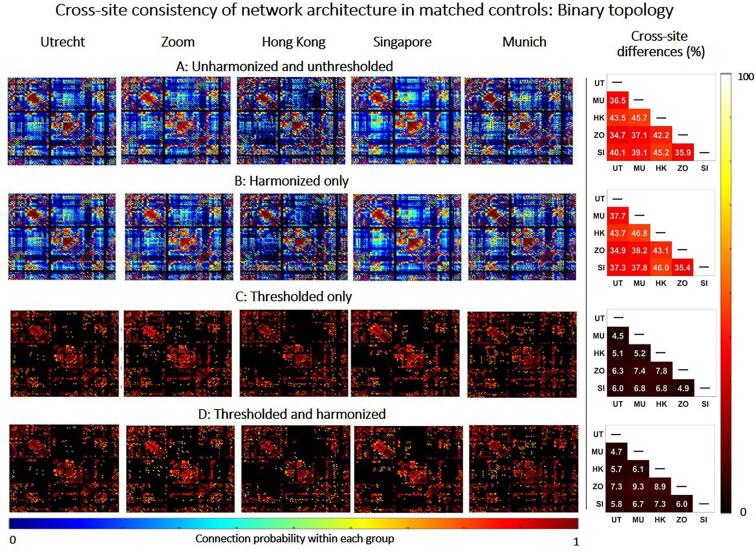
Consistency of network architecture (binary topology) between matched controls of different sites. Left: Connectivity matrices containing the probability of connections within each site (e.g., a connection with a probability of 0.2 is only detected in 20% of subjects of that group). The black background in the connectivity matrix means no connection is found between two nodes. Right: Relative difference between connectivity matrices of different sites. Results are displayed before harmonization and thresholding (A), after harmonization only (B), after thresholding only (C) and after combining the thresholding and harmonization (D).

Fig. 1 shows group-level connectivity matrices containing connection probability for age-matched controls of each site, before and after harmonization and thresholding. Before thresholding and harmonization, networks contained many connections with low probability (i.e., that were only reconstructed in a small proportion of subjects) (Fig. 1A). These low-probability connections were inconsistent and were reconstructed in different network locations, resulting in large differences in probability across sites (relative cross-site difference: 34.7 %–46.8 %, right panel). Harmonization alone did not change the probability of connections nor the consistency across sites (Fig. 1B). This implies that on average the same tracts were reconstructed before and after harmonization. This is illustrated in Supplementary Fig. S2, that shows a fibre bundle (e.g., corpus callosum) of one subject before and after harmonization, where the same streamlines were reconstructed. Thresholding had a significant impact on the connection probability and their consistency across sites since it removed low probability connections and retained connections with high probability (Fig. 1C and 1D). This resulted in more consistent network architectures across sites, with high-probability connections detected at the same network locations across sites (relative cross-site difference: 4.5 %–7.8 %). Since thresholding is by design applied after after tractography to create the connectivity matrices, it does not directly change the tractography results, but rather determines which edges (i.e. tracts) of the network are more consistently observed across subjects.

**Fig. 2:**
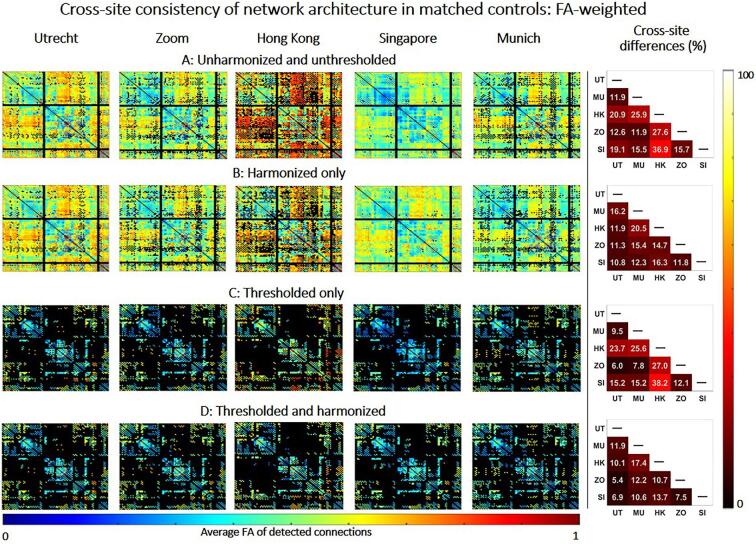
Consistency of network architecture (FA weight) between matched controls of different sites. Left: Connectivity matrix containing the average FA of connections detected within each site. The black background in the connectivity matrix means no connection is found between two nodes. Right: Relative difference between connectivity matrices of different sites. Results are displayed between before harmonization and thresholding (A), after harmonization only (B), after thresholding only (C) and after combining thresholding and harmonization (D).

Fig. 2 shows group-level FA-weighted connectivity matrices within each site, before and after harmonization and thresholding. FA values varied substantially across sites before harmonization, particularly for Hong Kong (relative cross-site difference up to 36.9 %, Fig. 2A). After harmonization, cross-site differences in FA across were minimized, even though the network still contained many low-probability connections (relative cross-site difference in FA: 10.8 %–20.5 %, Fig. 2B). Thresholding alone did not remove differences in FA across sites, because even though the connections retained were more consistent in probability, the weights of these connections still differed across sites due to scanner-related differences (Fig. 2C). When applied together, thresholding and harmonization produced networks that only contained high-probability connections with minimal differences in FA across sites (Fig. 2D). The voxel-wise harmonization of FA-values can be appreciated looking at a specific reconstructed and thresholded tract (e.g., the corpus callosum in Supplementary Fig. S3). In conclusion harmonization produced more similar FA values across sites without changing the reconstruction of tracts.

### 3.2 Sensitivity and precision to disease effects in SVD

**Fig. 3:**
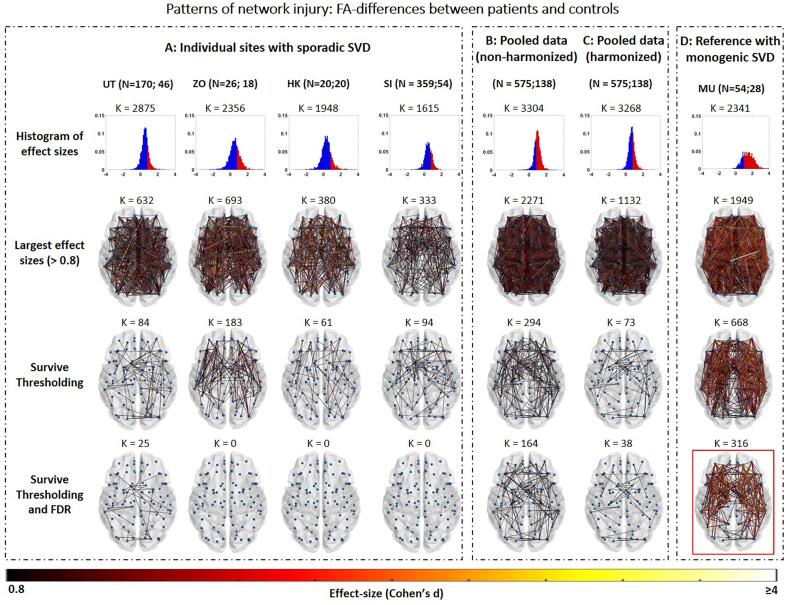
Patterns of network injury within sites with sporadic SVD versus controls (A), pooled data before (B) and after (C) harmonization as well as a reference cohort of CADASIL patients versus controls (D). N = x;y indicates numbers of patients and controls per site or in the pooled cohort. K represents the number of connections tested in the histogram or shown in the brain network map. First row: Histogram of effect sizes, showing Cohen’s d for all connections tested between patients and controls. Red areas represent effect sizes > 0.8. Second row: Connections with large effect sizes (>0.8) for patients compared to controls. Third row: Connections with large effect sizes that survive network thresholding. Third row: Connections with large effect sizes that survive thresholding and FDR correction. After each step the patterns of connections of each site were compared with the thresholded and FDR-corrected CADASIL cohort shown in red (bottom right). The ratio of true positives, false positives and precision relative to the CADASIL cohort were calculated and shown in Table 1. (For interpretation of the references to colour in this figure legend, the reader is referred to the web version of this article.)

Fig. 3 describes patterns of connections affected in patients relative to controls, with results shown for individual samples with sporadic SVD (A), pooled data of all sporadic SVD samples before (B) and after harmonization (C) as well as for the reference CADASIL sample (D). For individual sites with sporadic SVD, different disease burdens were observed across sites -reflecting differences in patient populations- as indicated by the range of effect sizes between patients and controls. Utrecht and Zoom had the most connections with large effect sizes, whereas Singapore and Hong Kong had the lowest disease burden (Fig. 3A, second row). However, in all sites the majority of strongly affected connections were likely spurious as they were only present in few patients and controls (i.e., connections with low probability at group level). When unthresholded connections were compared to those found in the reference CADASIL sample, a large proportion were false positives, which resulted in low precision within sites before thresholding (precision: 0.08–0.19, Table 1). Thresholding drastically reduced the number of false positives and improved precision within sites, relative to the reference (precision: 0.38–0.64, Fig. 3A, third row). However, only a small proportion of connections retained after thresholding also remained significant after FDR-correction (25 connections in the Utrecht cohort), indicating low sensitivity within individual sites (Fig. 3A, fourth row).

Regarding the pooled data before harmonization, effect sizes were inflated due to scanner-related differences in FA, resulting in >2/3 of all tested connections having large effect sizes (2271 connections with effect size > 0.8, Fig. 3B, first and second row). Moreover, the majority of these large effect sizes were found in low-probability connections, which resulted in low precision before thresholding, relative to the reference standard (precision: 0.06, Table 1). After thresholding, low probability connections were removed, resulting in improved precision (precision: 0.70, Table 1). Compared to individual sites, more connections survived FDR-correction in the non-harmonized pooled data (164 connections, Fig. 3B, fourth row), due to the larger sample size. After harmonization, the effect sizes observed in the pooled data were no longer inflated by acquisition-related differences and were closer to the range of effect sizes observed in individual samples with sporadic SVD (Fig. 1C, first and second row). Thresholding removed low probability connections, reducing the rate of false positives and improving precision (0.85). Sensitivity was apparently also improved since more connections survived FDR, as compared to individual sites (k = 38, Fig. 3C, fourth row).

## 4 Discussion

In a first study of its kind, using multicentre data of patients with SVD as an exemplar condition, we demonstrated that combining network thresholding and RISH harmonization improve consistency of dMRI-based brain networks across sites, while also increasing precision and sensitivity to detect connections affected in disease. Thresholding removed low probability connections improving the precision to detect connections most strongly affected within each cohort with sporadic SVD. Yet, individual sites showed low sensitivity to detect strongly affected connections, likely due to modest sample sizes, with only a small number of connections surviving FDR correction. Harmonization minimized cross-site differences in FA and enabled data pooling to achieve a large sample size and improve the sensitivity to detect strongly affected connections. When applied together, these two methods generated networks with more consistent sets of connections and more similar FA-weights across sites, while also achieving higher precision than individual sites.

### 4.1 Thresholding and harmonization improve network consistency

Preceding our work, several studies have analysed brain networks of older adults and the impact of SVD on global and local network properties as well specific patterns of injury (Du et al., 2020, Lawrence et al., 2018, Tuladhar et al., 2017). However, a frequently reported limitation in those studies is the low generalizability of results across datasets due to sample size or differences in network architecture (Smith et al., 2019). Our results indeed demonstrate that the network architecture before thresholding can differ substantially across age-matched controls with minimal burden of white matter injury. Within different sites, networks contained many unreliable tracts which were only reconstructed in a small proportion of subjects. These inconsistent tracts are likely a byproduct of cumulative errors during network reconstruction steps, especially in fiber tractography (de Reus and van den Heuvel, 2013a, de Reus and van den Heuvel, 2013b). These tracts had a wide-range of lengths (10–300 mm), indicating that thresholding was not biased to only short or long-range connections (see Supplementary Fig. S1). While no ground-truth brain network has yet been established, it is well accepted that connections that describe underlying white matter pathways should be detected consistently across subjects, with small degree of interindividual variation (Roberts et al., 2017). As shown by our current results as well as previous work, introducing thresholding as a step during network reconstruction removes low probability tracts and generates networks with more consistent architectures across datasets (Buchanan et al., 2020, Kurokawa et al., 2021, Messaritaki et al., 2019). We add to those results by showing that thresholding improves cross-site consistency in elderly subjects. We note that network thresholding is a higher-level step, since it is applied on the connectivity matrices and not on tractography itself. Therefore, it does not directly change the tractography results, but rather determines which edges of the network are more consistently observed across subjects. However the concept of thresholding (i.e., filtering noisy data) could also extend to other network reconstruction pipelines based on probabilistic tractography (Roberts et al., 2017). In this scenario, thresholding could be coupled with other established filtering strategies in probabilistic tractography (SIFT2, Smith et al., 2015). Of note, the same principles apply to functional connectomes where thresholding has been shown to increase edge-consistency across participants (Váša et al., 2017).

A possible concern about thresholding is that removing connections from the network can be detrimental to the sensitivity to disease effects (van Wijk et al., 2010). Although we cannot formally test sensitivity, because we have no standard of which connections were truly affected in each of the samples with sporadic SVD, it is arguably desirable to establish disease effects over a more consistent network architecture, less affected by spurious tracts (de Brito Robalo et al., 2020). In this work we applied a thresholding method that not only removes low probability connections across subjects, but also ensures that fixed network density is achieved across sites (van den Heuvel and Sporns, 2013). The stringency of thresholding remains arbitrary, but our own work and previous studies have shown that mid-range densities (≈15 %) reduces the number of false positives connections in networks reconstructed with deterministic fibre tractography, while maintaining the well-known small-world structure of brain networks and sensitivity to disease effects (Hagmann et al., 2008, Robalo et al., 2020, Roberts et al., 2017).

Our results also demonstrated that dMRI harmonization improves the consistency of connection weights across sites. Before harmonization, FA of reconstructed connections varied substantially between matched controls of different sites due to acquisition-related differences in the diffusion signal. Groups of healthy subjects that are matched for demographics should have similar diffusion profiles and therefore scalar diffusion metrics used for network weighting should not significantly differ across sites (Mirzaalian et al., 2015). RISH harmonization removed differences in the diffusion signal and produced networks with more similar FA across sites. The harmonization results shown in this study should be generalizable in other studies since it is applied at the beginning of the processing pipeline, prior to other methodological considerations. (e.g., FA, MD). Since the RISH method only requires a subset of matched subjects from each site as training subjects, it can be generalized to any multicentre cohort as long as training data is available. Importantly, researchers should be aware that subject matching is a challenging step, especially when dealing with elderly subjects or subjects with brain lesions. In those cases we recommend ensuring that brain lesions in the training data are comparable across sites, both in terms of location and severity.

While alternative methods for harmonization exist (e.g. statistical harmonization with ComBat, Fortin et al., 2017, mega and meta analysis), it remains unclear how to couple them with connectivity analysis where several network weights can be used and different graph metrics are derived for further analysis. Contrary to meta and mega-analysis which combine statistical results from already processed data (e.g., effect sizes), the RISH method aims at creating a single large dataset, allowing any subsequent analysis (such as network analysis or FADTTS). When the data is available, RISH harmonization should be prioritized over meta and mega-analysis. Here, we used FA-weighted networks, since this metric has been widely used as outcome in network studies of SVD to examine contrasts between patients and controls or association with cognition (Heinen et al., 2018, Reijmer et al., 2016). A recent report (Schilling et al., 2021) suggests that variability in scanner hardware and acquisition parameters is the largest source of uncertainty when performing fiber tractography with multi-site data. In our study, we did not observe large variability in brain networks before and after harmonization, which mainly impacted dMRI metrics of the connections, rather than their probability. This might imply that connection probability in brain connectivity is overall resistant to inter-scanner difference, especially when a relatively coarse-grained atlas is used to define the nodes (e.g. AAL atlas). On the other hand, data used in this work only included modest diffusion weightings, but the use of larger diffusion weightings – which is reccomended for accurate fiber tractography (Tournier et al., 2008) – might further exacerbate hardware-driven differences. Overall, when harmonization was combined with thresholding, the resulting networks had more consistent connections across subjects and across sites and more similar FA-weights. Overall, when harmonization was combined with thresholding, the resulting networks had more consistent connections across subjects and across sites and more similar FA-weights. A thresholded and harmonized network is thus a more reliable basis from which patterns of injury in SVD can be identified.

### 4.2 Thresholding and harmonization improve sensitivity and precision to effects of interest

In the second part of this study, we examined the impact of thresholding, harmonization and pooling on the sensitivity and precision to detect connections strongly affected in SVD. Our findings within sporadic SVD samples show that before thresholding there is low precision to detect relevant connections compared to the reference standard. This is in part due to the fact that large effect sizes were detected in low probability connections, which were absent in a large proportion of subjects, and likely represent artefacts. For these low probability connections, the number of data points used for the t-tests in the NBS pipeline is even smaller than the already small sample size within sites (number of patients and controls). This resulted in many outlier connections showing large effect sizes (>0.8). Thresholding removed a large proportion of false positive connections, ensuring that all large effect sizes were identified only for connections that were present in a large proportion of subjects (i.e., the actual sample size used for the *t*-test was closer to the group sample size). This resulted in higher precision to detect strongly affected connections. This finding is in line with previous research with healthy subjects from the UK biobank, which showed that connections retained in the network after thresholding are more strongly associated with biological effects of interest, such as age (Buchanan et al., 2020).

Within each site, sensitivity was very low since few connections remained significant after FDR-correction. Significant connections were only retained in the Utrecht cohort, which had relatively high disease burden and a substantial sample size. In all other sites, no significantly different connection between patients and controls remained after FDR, likely due to limited sample size. For example, the Zoom Cohort had effect sizes relatively similar to Utrecht. For example, the Zoom Cohort had relatively similar effect sizes as Utrecht, but a limited sample small size to detect significant connections. On the other hand, Singapore had the largest sample size from all sites but since patients were community-duelling individuals with lower disease burden and more subtle effect sizes, but no connection survived FDR. So far, most network analysis studies in SVD employed limited sample sizes, with the exception of a recent work with 930 subjects that identified key connections disrupted in SVD, comprising interhemispheric connections as well as connections between subcortical and frontal brain regions (Petersen et al., 2020). An application of network localisation of SVD-related injury could be to help understanding how diffuse and/or focal damage in certain brain areas affects cognitive function (Biesbroek et al., 2018). If these findings are to be translated in more applied settings, they should be validated with large multicentre datasets and generalized across centers. With our pooled analysis we demonstrated that combining multicentre data to achieve a larger sample size can improve the sensitivity to detect connections affected in SVD, which would not have been possible using single center data. Importantly, our results clearly show that this should only be performed after harmonization to avoid introducing bias in effect sizes, in analogy to previous studies on diffusion tensor imaging metrics (Cetin-Karayumak et al., 2020, de Brito Robalo et al., 2021). When the data was pooled without harmonization, effect sizes were much larger than those observed within individual sites, which is likely erroneous. Consequently, >2/3 of all the tested connections had an effect size >0.8, which originates from acquisition-related differences in FA across sites, combined with imbalance in proportions of controls and patients in the samples. Even though more “affected connections” were detected, these were mostly false positives, resulting in low precision. Of note, without harmonization, the opposite scenario could also be possible, i.e., where pooling data results in a loss of effect size rather than inflation, depending on patient and control sampling and whether scanner effects produce higher or lower FA values for one site, relative to another. To avoid these detrimental scenarios, it is crucial that harmonization is performed prior to data pooling to ensure that the detected differences are not driven by acquisition-related differences across sites but by the increased power of a larger sample size. In summary, when applied together, thresholding, harmonization and pooling improved sensitivity to detect connections affected in sporadic SVD with higher precision than individual sites.

## 5 Limitations and future outlook

A limitation of our study is the lack of a ground-truth for patterns of injured connections in SVD. By defining the CADASIL sample as model for patterns of network injury in SVD, we created a reference standard to compare the findings in patients with sporadic SVD from each site to. We realize that patterns of injury observed in CADASIL do not represent an actual ground-truth of network injury in sporadic SVD, but rather represent a typical example of most severely affected tracts to support our analyses. Using this reference standard, we focused on improving processing steps to identify network injury. Of note, at this stage we did not aim to obtain novel insight into actual SVD disease effects. Accordingly, we did not harmonize patient selection for particular disease stages, risk profiles, or outcomes. Our selection was primarily based on white matter lesion burden. Future studies could use the techniques demonstrated here to further characterize the actual patterns of disrupted connections in patients with SVD and their functional impact. This should also entail more rigorous selection of patients and controls, considering parameters such as risk factors, comorbidities or clinical diagnosis (e.g., mild cognitive impairment; dementia). Furthermore, recently developed techniques such as machine learning and classification algorithms could benefit from large sample sizes gained by harmonizing multicentre data. In future studies it could be of interest to use classifiers to determine which patients are likely to have cognitive decline over time based on network metrics or which white matter tracts can predict change in specific cognitive domains. For such studies to be feasible it is important to have a large test sample to train the classifier and to perform validation.

Our study was also limited to datasets with lower quality (e.g., lower angular resolution) as compared to other connectivity studies in healthy young adults (Sotiropoulos et al., 2013), but on the other hand reflects the reality in a clinical setting. Another point to consider from a technical perspective, is that network reconstruction involves many methodological choices which could not all be tested in this study, and are topics of debate (e.g., registration methods, tractography algorithm, parcellation schemes, Yeh et al., 2021). Finally, we examined network consistency based on primary features of network architecture (e.g., connection probability and connection weight), but topological metrics derived from graph theory should also be investigated in future analyses (Bullmore and Sporns, 2009).

## 6 Conclusion

Using multicentre dataset of patients with SVD, we demonstrated that sensitivity and precision to detect disease effects in specific network connections can be improved by thresholding, harmonization and pooling. Future studies that investigate network localisation of SVD-related injury, or other diseases, and associations with brain function should introduce thresholding as part of the processing pipeline, to ensure that disease effects are evaluated over a network with a more consistent architectures and fewer positives. Furthermore, since effect sizes are likely to be subtle, large sample sizes should be used to study these effects. We provide proof of concept that harmonization facilitates pooling of large multicentre datasets to achieve this goal.

## 7 Data availability statement

The data used in this study is available upon contact and agreement with the respective investigators of each cohort.

## Study funding

This work was supported by ZonMw, The Netherlands Organisation for Health Research and Development (VICI grant 91816616 to G.J. Biessels). The research of A. Leemans is supported by VIDI Grant 639.072.411 from the Netherlands Organization for Scientific Research (NWO). Zoom@SVDs is part of the SVDs@target project. SVDs@target has received funding from the European Union’s Horizon2020 research and innovation program under grant agreement No 666881. The CUHKRI is supported by General Research Fund (Grant No GRF CUHK 471911), the Lui CheWoo Institute of Innovative Medicine, and Therese Pei Fong Chow Research Centre for Prevention of Dementia (in memory of Donald H. K. Chow). Funding for the EDIS study was provided by the National Medical Research Council of Singapore.

## CRediT authorship contribution statement

**Bruno M. de Brito Robalo:** Conceptualization, Data curation, Formal analysis, Investigation, Investigation, Methodology, Project administration, Resources, Software, Validation, Visualization, Writing – original draft, Writing – review & editing. **Alberto de Luca:** Conceptualization, Funding acquisition, Methodology, Supervision, Validation, Visualization, Writing – review & editing. **Christopher Chen:** Data curation, Funding acquisition, Investigation, Writing – review & editing. **Anna Dewenter:** Data curation, Funding acquisition, Investigation, Writing – review & editing. **Marco Duering:** Data curation, Funding acquisition, Investigation, Writing – review & editing. **Saima Hilal:** Data curation, Funding acquisition, Investigation, Writing – review & editing. **Huiberdina L. Koek:** Data curation, Funding acquisition, Investigation, Writing – review & editing. **Anna Kopczak:** Data curation, Funding acquisition, Investigation, Writing – review & editing. **Bonnie Yin Ka Lam:** Data curation, Funding acquisition, Investigation, Writing – review & editing. **Alexander Leemans:** Data curation, Funding acquisition, Investigation, Writing – review & editing. **Vincent Mok:** Data curation, Funding acquisition, Investigation, Writing – review & editing. **Laurien P. Onkenhout:** Data curation, Funding acquisition, Investigation, Writing – review & editing. **Hilde van den Brink:** Data curation, Funding acquisition, Investigation, Writing – review & editing. **Geert Jan Biessels:** Conceptualization, Funding acquisition, Methodology, Supervision, Validation, Visualization, Writing – review & editing.

## Declaration of Competing Interest

The authors declare that they have no known competing financial interests or personal relationships that could have appeared to influence the work reported in this paper.

## Data Availability

Data will be made available on request.
