# Supplementary material for: Improved sensitivity and precision in multicentre diffusion MRI network analysis using thresholding and harmonization
Source: Neuroimage Clin. 2022 Oct 3;36:103217. doi: 10.1016/j.nicl.2022.103217 (PMC9668636; doi:10.1016/j.nicl.2022.103217)
Supplement: Supplementary data 1 [file mmc1.docx]

**Table 1:** Demographics and imaging parameters of the study samples.

|  | Utrecht 1 | | Utrecht 2 (ZOOM) | | Hong Kong | | Singapore | | Munich | |  |
| --- | --- | --- | --- | --- | --- | --- | --- | --- | --- | --- | --- |
|  | Controls  (N = 46) | Patients  (N = 170) | Controls  (N = 18) | Patients  (N = 26) | Controls  (N = 20) | Patients  (N = 20) | Controls  (N = 54) | Patients  (N = 359) | Controls  (N = 28) | Patients  (N = 54) |  |
| Demographics |  |  |  |  |  |  |  |  |  |  |  |
| Age, years | 71.2 ± 4.8 | 74.9 ± 9.0 | 62.4 ± 6.9 | 64.8 ± 6.8 | 69.2 ± 3.4 | 74.1 ± 3.3 | 66.6 ± 4.8 | 71.3 ± 6.0 | 71.8 ± 7.4 | 56.0 ± 7.1 |  |
| Male sex (%) | 28 (61) | 99 (59) | 10 (55) | 18 (69) | 10 (50) | 10 (50) | 31 (52) | 166 (46) | 16 (57) | 20 (37) |  |
| MRI markers |  |  |  |  |  |  |  |  |  |  |  |
| WMH volume | 0.3 [0.1, 0.6] | 1.2 [0.5, 2.7] | 0.03 [0.02, 0.08] | 0.7 [0.3, 1.3] | 0.1 [0.05, 0.1] | 0.6 [0.3, 1.1] | 0.04 [0.02, 0.09] | 0.8 [0.08, 1.16] | 0.2 [0.1 0.7] | 6.2 [3.5, 10] |  |
| WMH (Fazekas) | 0 [0, 1] | 2 [1, 2] | 1 [1,1] | 2 [2,3] | 0.5 [0, 1] | 2 [2, 3] | 1 [1,1] | 2 [1,3] | 0 [0, 1] | 3 [2, 3] |  |
| Lacunes (% present) | 0 (0) | 69 (36) | 0(0) | 12 (46) | 0 (0) | - | 0 (0) | 118 (33) | 0 (0) | 40 (74) |  |
| Imaging parameters |  |  |  |  |  |  |  |  |  | |  |
| Scanner | 3T Philips Achieva | | 3T Philips Achieva | | 3T Philips Achieva | | 3T Siemens Magnetom Trio, Tim | | 3T Siemens Verio | |  |
| Software version | R3.1 | | MR Release 5.6.0 | | MR Release 5.1 | | syngo MR B19 | | syngo MR B19 | |  |
| Voxel size (mm^3^) | 2.5 × 2.5 × 2.5 | | 2.5 × 2.5 × 2.5 | | 1 × 1 × 2 | | 3.1 × 3.1 × 3.0 | | 2 × 2 × 2 | |  |
| b-value (s/mm^2^) | 1200 | | 1200 | | 1000 | | 1150 | | 1000 | |  |
| # of directions | 45 | | 45 | | 32 | | 61 | | 30 | |  |

Data presented as mean ± SD, number (percentages) or median [interquartile range]; SVD = small vessel disease; CADASIL = cerebral autosomal-dominant arteriopathy with subcortical infarcts and leukoencephalopathy; WMH = white matter hyperintensity (normalized to % of intracranial volume).


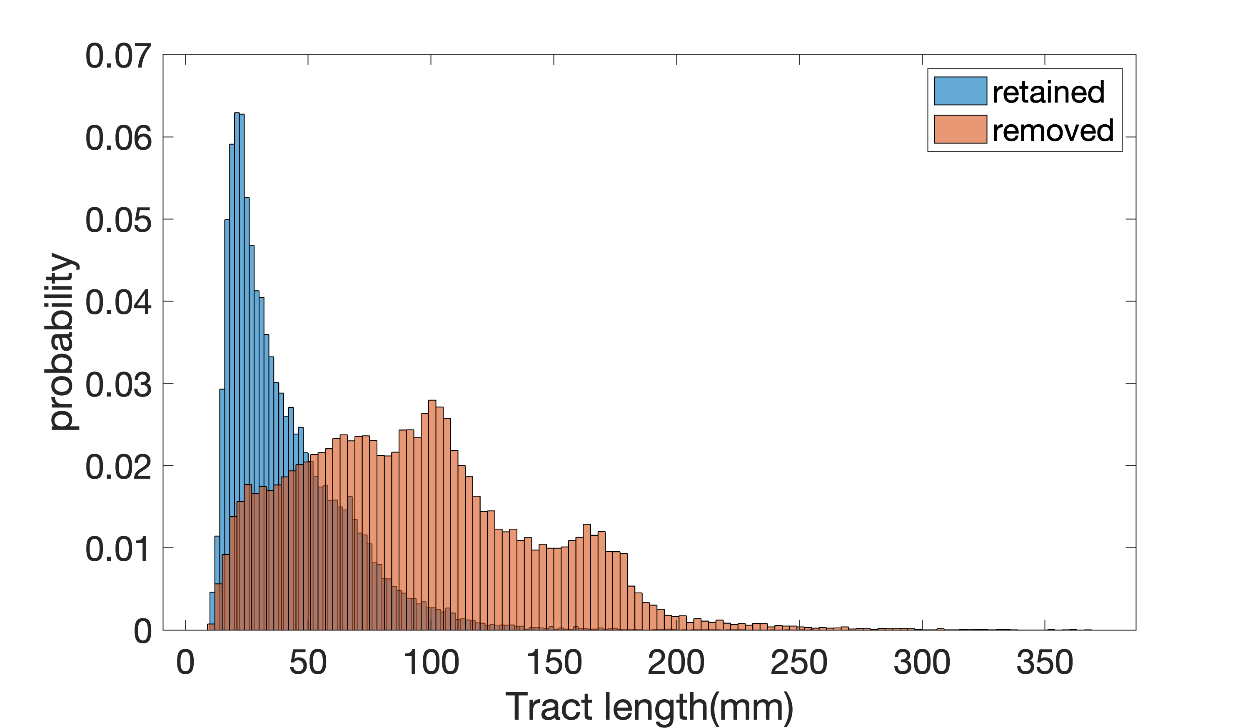


**Figure S1:** Average tract length distribution of connections retained and connections removed from the network during thresholding.


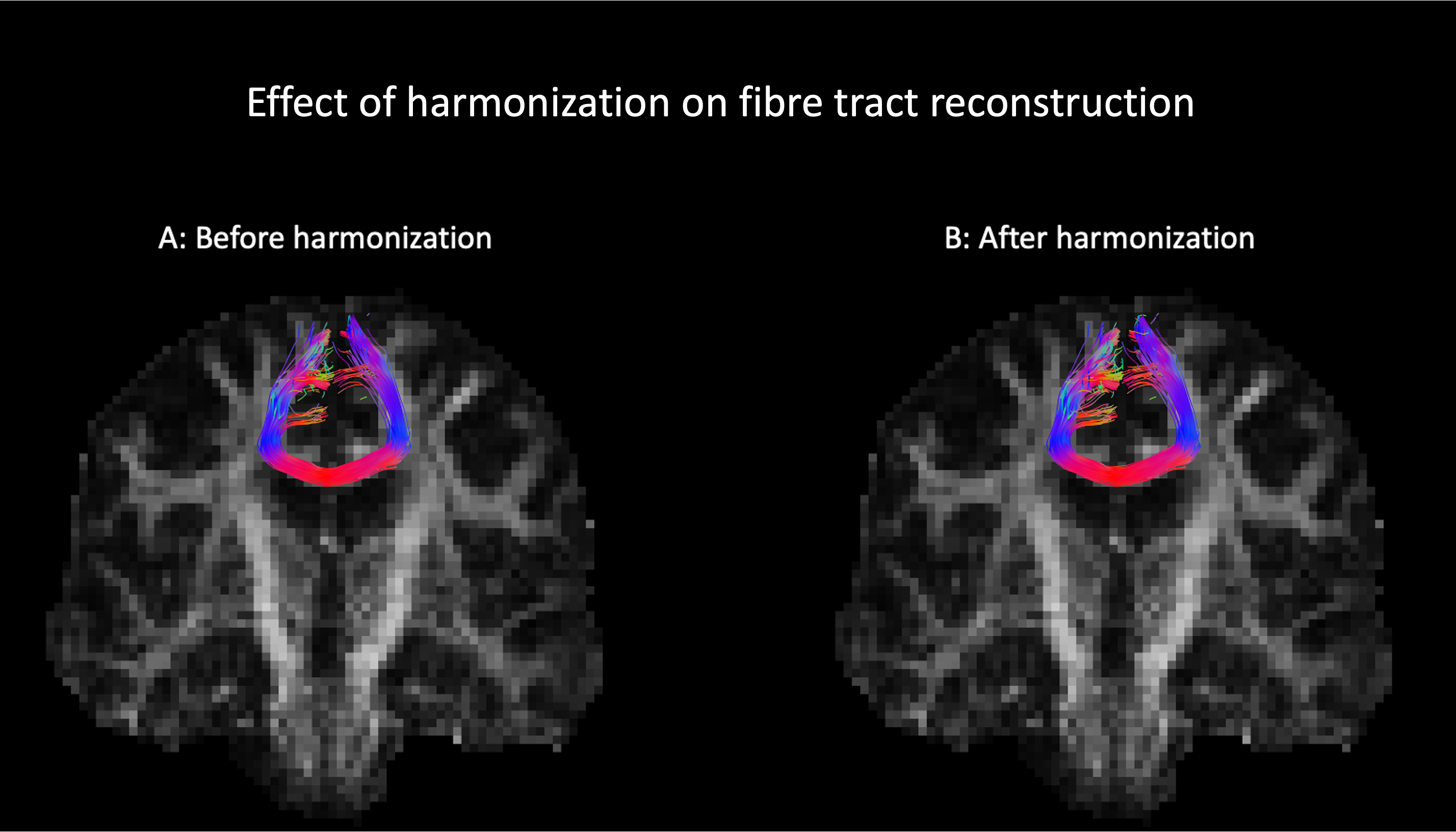


**Figure S2:** Fiber tractography of the corpus callosum before (A) and after (B) harmonization of an example control subject. The figure shows that harmonization does not change the reconstruction of fiber tracts, nor their pathways. Color coding: red (left-right); blue (superior-inferior); green (anterior-posterior). This supports our main results in Figure 1 where we observed that harmonization alone does not affect the probability of a connection being reconstructed. Harmonization can affect the metrics of the reconstructed connection though (see Figure S3).


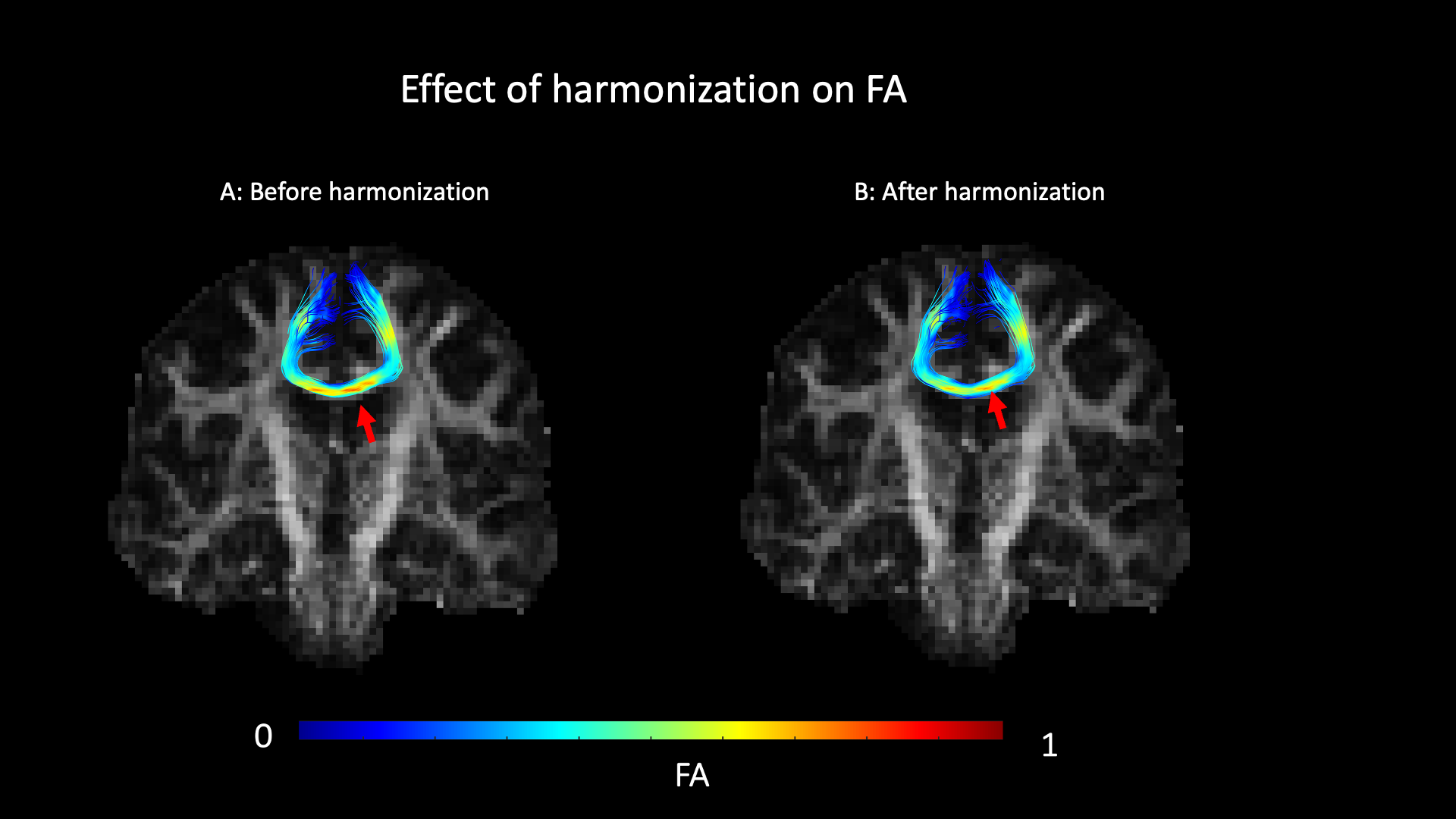


**Figure S3:** Fiber tractography of the corpus callosum before (A) and after (B) harmonization of an example control subject, color-coded by FA. The arrows indicate regions along the tract where FA changes after harmonization. This is in line with our main results in Figure 2, where we observed that the FA-weighted connectivity matrix is scaled after harmonization and becomes more similar across sites.
